# AI-based detection of lung lesions in [^18^F]FDG PET-CT from lung cancer patients

**DOI:** 10.1186/s40658-021-00376-5

**Published:** 2021-03-25

**Authors:** Pablo Borrelli, John Ly, Reza Kaboteh, Johannes Ulén, Olof Enqvist, Elin Trägårdh, Lars Edenbrandt

**Affiliations:** 1 Department of Clinical Physiology, Sahlgrenska University Hospital, Gothenburg, Sweden; 2 Department of Radiology, Kristianstad Hospital, Kristianstad, Sweden; 3 Department of Translational Medicine and Wallenberg Center for Molecular Medicine, Lund University, Malmö, Sweden; 4 Eigenvision AB, Malmö, Sweden; 5 Department of Electrical Engineering, Chalmers University of Technology, Gothenburg, Sweden; 6 Department of Clinical Physiology and Nuclear Medicine, Skåne University Hospital, Malmö, Sweden; 7 Department of Molecular and Clinical Medicine, Institute of Medicine, Sahlgrenska Academy, University of Gothenburg, Gothenburg, Sweden

**Keywords:** AI, FDG, PET-CT, Lung cancer, Segmentation, Automatic, Total lesion glycolysis

## Abstract

**Background:**

[^18^F]-fluorodeoxyglucose (FDG) positron emission tomography with computed tomography (PET-CT) is a well-established modality in the work-up of patients with suspected or confirmed diagnosis of lung cancer. Recent research efforts have focused on extracting theragnostic and textural information from manually indicated lung lesions. Both semi-automatic and fully automatic use of artificial intelligence (AI) to localise and classify FDG-avid foci has been demonstrated. To fully harness AI’s usefulness, we have developed a method which both automatically detects abnormal lung lesions and calculates the total lesion glycolysis (TLG) on FDG PET-CT.

**Methods:**

One hundred twelve patients (59 females and 53 males) who underwent FDG PET-CT due to suspected or for the management of known lung cancer were studied retrospectively. These patients were divided into a training group (59%; *n* = 66), a validation group (20.5%; *n* = 23) and a test group (20.5%; *n* = 23). A nuclear medicine physician manually segmented abnormal lung lesions with increased FDG-uptake in all PET-CT studies. The AI-based method was trained to segment the lesions based on the manual segmentations. TLG was then calculated from manual and AI-based measurements, respectively and analysed with Bland-Altman plots.

**Results:**

The AI-tool’s performance in detecting lesions had a sensitivity of 90%. One small lesion was missed in two patients, respectively, where both had a larger lesion which was correctly detected. The positive and negative predictive values were 88% and 100%, respectively. The correlation between manual and AI TLG measurements was strong (*R*^2^ = 0.74). Bias was 42 g and 95% limits of agreement ranged from − 736 to 819 g. Agreement was particularly high in smaller lesions.

**Conclusions:**

The AI-based method is suitable for the detection of lung lesions and automatic calculation of TLG in small- to medium-sized tumours. In a clinical setting, it will have an added value due to its capability to sort out negative examinations resulting in prioritised and focused care on patients with potentially malignant lesions.

## Background

Characterisation of lung lesions has become one of the main indications for [^18^F]-fluorodeoxyglucose (FDG) positron emission tomography with computed tomography (PET-CT) in recent years in nuclear medicine and radiology departments [1]. Comprehensive guidelines for the management of lung nodules produced by The American College of Chest Physicians, British Thoracic Society and Fleischner Society all recommend PET-CT in nodules > 8 mm with moderate (5–65%) pre-test probability of malignancy [2–4]. In reality, it has been reported that clinicians use PET-CT assertively to investigate nodules with low risk (< 5%) of malignancy even though guidelines do not recommend it [5]. A study has demonstrated that a critical number of patients with lung nodules and considered low risk had positive PET-CT findings and proven malignant histological diagnosis [6].

On the opposite side in the characterisation of larger lung lesions, PET-CT has become the tool of choice diminishing risks for patients from invasive techniques [7, 8] and also providing valuable theragnostic tumour information [9] and tumour texture analysis [10].

Recently, Sim et al. suggested that AI can improve nodules detection efficacy by radiologists in chest radiographs [11]. For PET-CT studies, most of the research has been done in automatically obtained theragnostic [9] and radiomic features but always with manual tumour localisation.

Automatic lung lesion segmentation [12] has been proposed and done with success but as aforementioned the segmentations were aided by manual tumour localisation. A promising work done by Schwyzer et al. [13] was able to correctly characterise cancer or not cancer using general class activation maps but not specific lesion localisation. Furthermore, Kirienko et al. were able to correctly identify T-stage using convolutional neural network (CNN) but also using manually located known tumours in the lungs [14]. The most recent success in utilising AI fully automatically to localise and classify abnormal FDG uptakes has been demonstrated by Sibille et al. [15]. To our knowledge, the investigation of combining automatic segmentation and extraction of radiomic data from lung lesions using AI has not been done before.

Total lesion glycolysis (TLG) is an emerging imaging biomarker which is calculated by multiplying the metabolic tumour volume with SUV_mean_. For non-small cell lung cancer, it has been shown that TLG can be used to prognosticate progression-free survival and overall survival [16, 17] and as an early post-treatment predictor of response [18–20].

Our aim was to develop a completely automated method based on AI for the analysis of FDG PET-CT in patients with known or suspected lung cancer and measure the TLG compared to manual measurements.

## Material and methods

**Fig. 1:**
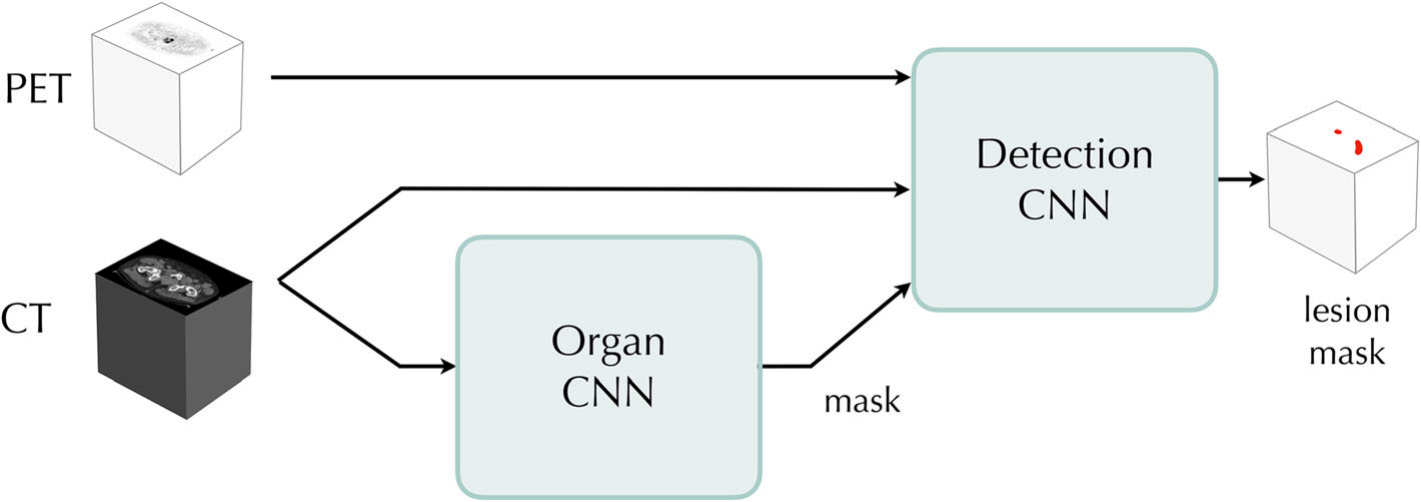
Schematic design of the AI-based tool. There are three inputs to the Detection CNN, the PET image, the CT image and an organ mask generated by the Organ CNN. The output from the Detection CNN consists of lesion probabilities that can be thresholded to produce a lesion mask

The AI-based tool consists of two CNNs, the Detection CNN trained to detect lung lesions and the Organ CNN trained to segment organs. A mask of the organs segmented by the Organ CNN are used as an auxiliary input to the Detection CNN (Fig. 1).

### Patients

For training and evaluating of the Detection CNN, images from a total of 112 patients were recruited retrospectively. These were patients who underwent clinically indicated FDG PET-CT due to suspected lung cancer or for the management of known lung cancer between April 2008 and December 2010. In the selection process, three patients were excluded because of centrally located tumour, likely sarcoid disease and mediastinal tumour. The patients had a mean age of 65.3 years (range 43–85) of which 59 were females and 53 males. The patient group was divided into a training group (59%; *n* = 66), a validation group (20.5%, *n* = 23) and a test group (20.5%; *n* = 23).

### Imaging

PET-CT data were obtained using an integrated PET-CT system (Siemens Biograph 64 Truepoint). The patients were injected with 4 MBq/kg (maximum of 400 MBq) of FDG and fasted for at least 4 h prior to the injection. The accumulation time was 60 min. Images were acquired with 3 min per bed position from the base of the skull to the mid-thigh. PET images were reconstructed with a slice thickness of 3 mm and pixel spacing of 4.07 mm with an iterative ordered subset expectation maximisation 3D algorithm (four iterations, eight subsets), matrix size 168 × 168. CT-based attenuation and scatter corrections were applied. A low-dose CT scan (64-slice helical, 120 kV, 30 mAs, 512 × 512 matrix) was obtained covering the same part of the patient as the PET scan with slice thickness of 3 mm and pixel spacing of 1.37 mm. The CT was reconstructed using a filtered back projection algorithm with slice thickness and spacing matching the PET scan.

### AI-model

**Fig. 2:**
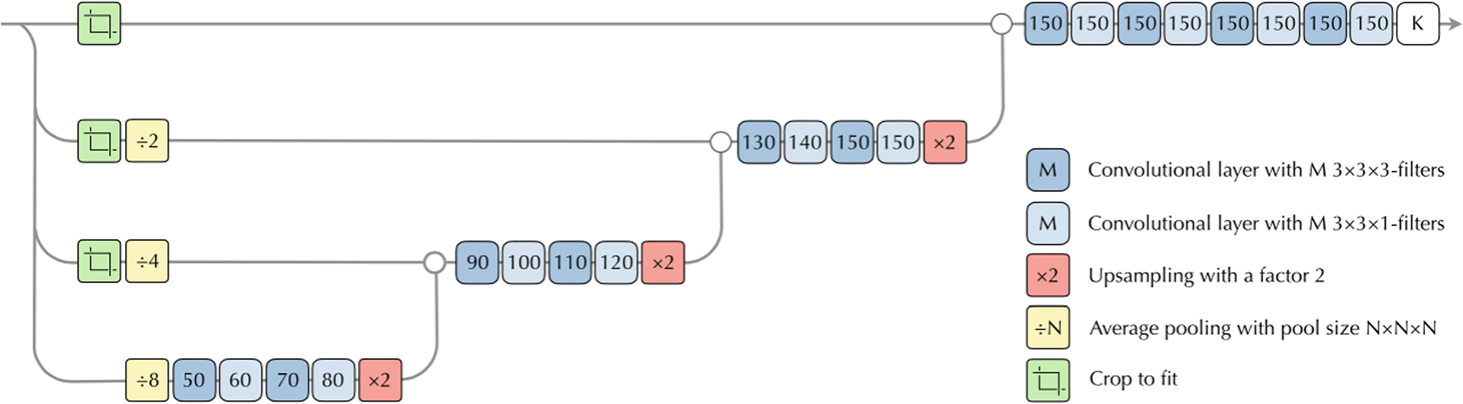
Structure of the networks. Due to the pooling layers the network works on four different resolutions. This allows a large receptive field at low memory cost during training. All convolutional layers use rectified linear unit activations apart from the last one using a softmax activation to produce the final output probabilities

As mentioned above, our AI model was based on two convolutional networks. The Organ CNN from [21], segments a number of different organs and inputs an organ mask marking the lungs, vertebral bones, liver, aorta and the heart to the Detection CNN. Apart from this, the Detection CNN also takes the PET and CT images as input (Fig. 1). To simplify the structure, the PET image was resampled to the CT resolution. Combining this information, the Detection CNN tries to classify each image voxel as either background or lung lesion. The Detection CNN has the network architecture from [21] except that it takes a multi-channel input consisting of the PET image, the CT image and the organ mask previously described. The structure of the CNN is shown in Fig. 2.

#### Training the Detection CNN

The Detection CNN was trained using the training set (66 images, with 74 lesions in total) for direct parameter estimation and the validation set (23 images, with 35 lesions in total) to choose learning rate. Since exact delineation of lesions is virtually impossible, mimicking the exact boundaries of the annotations is not relevant. Thus, any voxels within 10 mm from the annotated lesions are marked as “don’t-care”. This means that when computing the loss function, there is no loss for these voxels regardless of the output label. For the remaining voxels, the standard negative log-likelihood loss was used. Naturally, this leads to a slight over-segmentation of the lesions, but as detection is the main goal here, this was considered acceptable.

The optimization was performed using the Adam method with Nesterov momentum. The learning rate was initialized at 0.0001 and reduced when the validation loss reached a plateau. Each epoch consisted of 500 batches each containing 75 patches; the patch size is smallest possible for the network, 136 × 136 × 72 pixels. After 50 epochs, the model was evaluated on the training group. Patches whose center points were classified as false positives were sampled more frequently (10% of the samples) when the training was restarted. This cycle was repeated ten times.

#### Postprocessing

The Detection CNN was only applied to pixels between the top of the lungs and the bottom of the lungs (determined by the Organ CNN). To reduce noise, all connected components smaller than 1 mL in volume was removed. Finally, a blacklist mask was created from the organ mask (excluding the lungs, and with a 10 mm dilation of the heart). Using a watershed transform, each voxel was associated to a local SUV_max_ in the PET image. If this SUV_max_ belonged to the mask, the voxel was excluded.

### Manual segmentation

A cloud-based annotation tool (RECOMIA, https://www.recomia.org) was used. Reader A, a nuclear medicine specialist, made segmentations in the tool with minor adjustments by a radiology resident with experience in segmentations which were agreed upon. These annotations were appointed as ground truth and used in training the CNN (training and validation set) and evaluated (test set). Two additional nuclear medicine specialists, reader B and C, made separate annotations in the test set for comparison with the ground truth and AI-model, respectively. The additional annotations by reader B and C were not used for training of the CNN. Abnormal lung lesions with increased FDG-uptake in the fused PET-CT images were segmented using a built-in freehand tool.

### Statistical methods

Sensitivity was calculated on a lesion level, whereas specificity was left out due to the known problem of defining meaningful true-negative samples in this kind of study. Dice index was calculated to evaluate the agreement between readers and the AI-model. TLG was calculated for every lung lesion in each of the 15 patients with the following formula: TLG = metabolic tumour volume x mean standard uptake value (SUV_mean_) of the lesion. A Bland-Altman analysis was used to visually assess the level of agreement between automatic and manual TLG measurements. Correlation between manual and AI-based TLG was assessed using Pearson correlation coefficient.

## Results

**Fig. 3:**
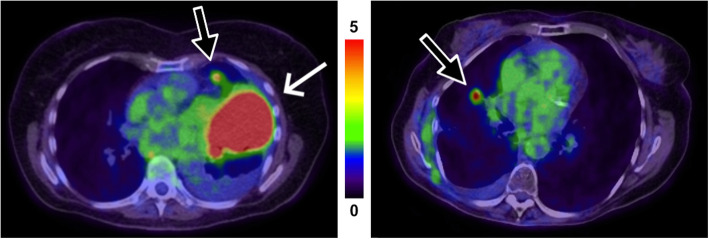
Two patients (left and right respectively), each with a missed lung lesion (black arrow) by the AI-model. Both were less than 1 mL and therefore removed in the post-processing. The larger lesion in the left image (white arrow) was detected correctly. Segmentations are not shown

Eight of the 23 patients in the test group showed no lung lesion. The remaining 15 patients had a total of 20 lung lesions. The AI-based tool detected 18 of these 20 lesions (90% sensitivity). One small lesion adjacent to the heart border was not detected (false negative) in a patient who also had a large lung lesion correctly detected by the AI-based tool. The other missed lesion was located in the right hilar region in a patient with a larger apical lesion in the ipsilateral lung. The two missed lesions are shown in Fig. 3. The AI-based tool detected 7 regions of false positive regions where 3 of these were in 2 true negative patients and the rest (4 segmentations) in 3 other patients.

On a patient level, the positive and negative predictive values for lung lesions were 88% and 100%, respectively.

Lesion agreement, the ability to segment the same lesion regardless accuracy, is summarised in Table 1. Dice index was on average 0.75, 0.71 and 0.49 when reader A was compared with reader B, C and the AI-model, respectively (Table 2).

**Table 1:** Lesion agreement. Three readers independently annotated lung cancer lesions in the test images. The lesions where defined using the union of all readers’ manual annotations. The less number of readers that agree on a lesion, the more difficult it can be considered. The CNN missed 2 lesions that all readers agreed on, and marked 7 regions that no reader marked

Number of readers agree	Count	Reader A	Reader B	Reader C	CNN
**3**	18	18	18	18	16
**2**	1	1	1	0	1
**1**	9	1	5	3	3

**Table 2:** Dice index of lesions (reader A was appointed as ground truth)

	Reader B	Reader C	CNN
**Average**	0.75	0.71	0.49
**Min**	0.0	0.0	0.0
**Max**	0.92	0.92	0.87

**Fig. 4:**
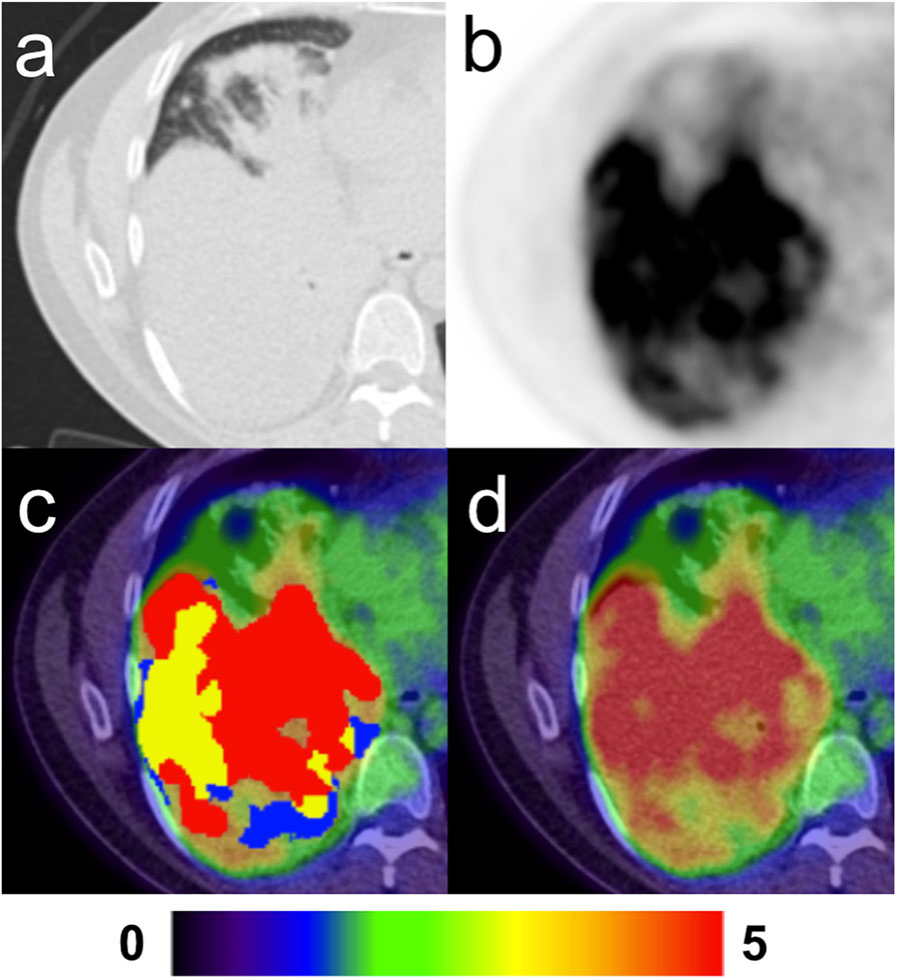
Large lesion with complex necrosis in the right lung. **a** Axial CT with lung window. **b** Axial PET. **c** Fused axial PET-CT with overlaying segmentations; manual only (red), AI-only (blue) and manual + AI (yellow). **d** Fused axial PET-CT

In one case, a patient with a large lung tumour that occupied most of the right lower lung (Fig. 4) showed apparent difficulty for the AI-model to segment closely to the ground truth. The lesion was the largest one in the test sample and highly irregular in FDG-avidity due to complex necrotic areas. Detection by the AI-model of such lesions is evidently not a problem but can pose difficulties in accurate volume and SUV_mean_ measurements.

**Fig. 5:**
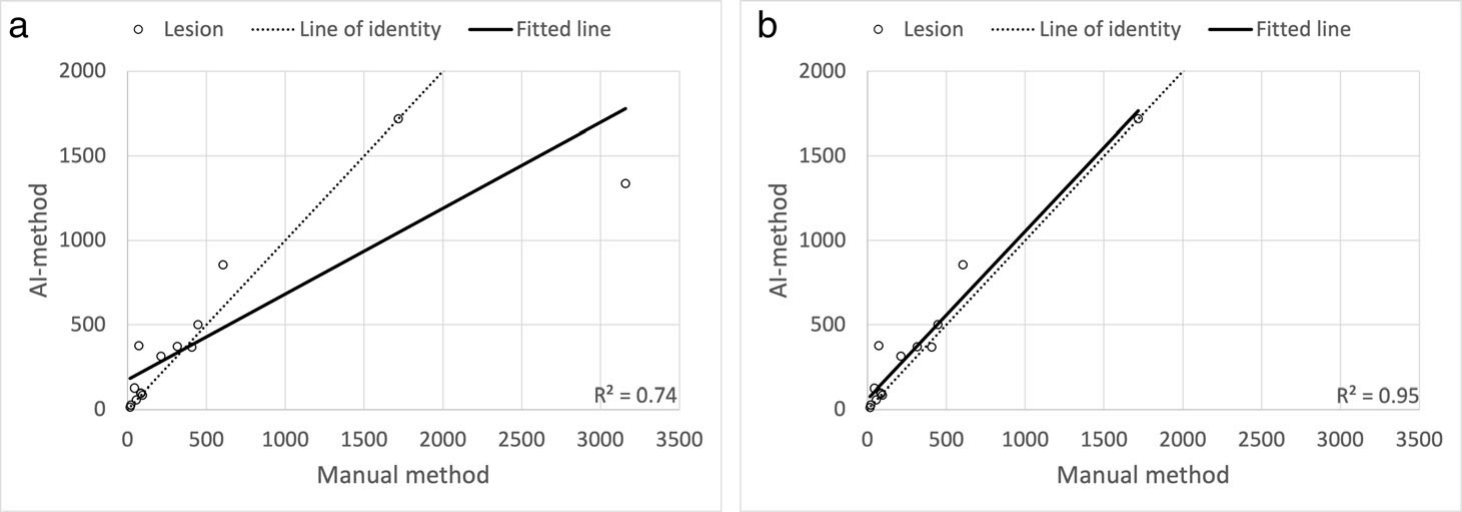
**a** Scatter plot showing manually measured TLG plotted against measurements made by the AI-method in the test group. The largest measured data point show much greater difference between the two methods compared to the smaller measurements; analysis of this individual lesion shows different morphology and localisation adjacent to the heart which caused underestimation with the AI-method (42% of ground truth). This may indicate that the AI-method underperforms in large complex lesions that are adjacent to the heart. **b** Scatter plot showing manually measured TLG plotted against measurements made by the AI-method in the test group with removed outlier described in (**a**)

**Fig. 6:**
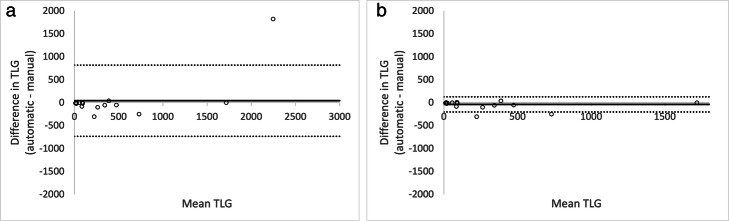
**a** Bland-Altman analysis of TLG differences between AI and manual methods of abnormal lung lesions in the test group. The mean was 42 g, 95% limits of agreement were − 736 g to 819 g. **b** Bland-Altman analysis of TLG differences between AI and manual methods of abnormal lung lesions in the test group with removed outlier described in Fig. 5a. The mean was − 39 g, 95% limits of agreement were − 204 g to 125 g

Scatter plots showing the relation between manually and automatic measured TLG is shown in Fig. 5a, b. There was strong correlation (*R*^2^ = 0.74) and very strong correlation (*R*^2^ = 0.95) when the outlier was removed, respectively between the measurements. The Bland-Altman analysis is shown in Fig. 6a, b. The two methods showed good agreement for most of the tumours, lesions with mean TLG < 200 in particular. There was a considerable TLG discrepancy in the large tumour with complex necrosis mentioned before, which caused the wide limits of agreement, − 736 to 819 g. In Fig. 6b, this patient was removed from the Bland-Altman analysis which results in narrower limits of agreements, − 204 to 125 g.

## Discussion

On a lesion-based analysis, our AI-based tool was able to identify 90% of the lesions, missing just two small lesions in patients where the primary tumour was correctly detected. Therefore, the AI method did not incorrectly miss any patients with lesions (100% negative predictive value on a patient-basis) which is probably the most promising feature extracted in this study and could probably be the first main indication for this type of AI-tool. Furthermore, the missed lesions were due to the post-processing step where lesions less than 1 mL were removed, which is a limitation of the study. The volume limit was chosen based on the validation set; it removed a few small false positive detections while keeping all true positive components (for the validation set). In a clinical setting where each finding is manually considered, this threshold can be lowered. In a screening scenario, an arbitrary volume limit could be set to match one of the many guidelines regarding solitary pulmonary nodules [2–4]. If the purpose is to maximize sensitivity at the cost of false positives, positive predictive value and irritation of the physician, then lowering the limit or even abolishing it is preferred. An ideal AI-model would have sensitivity appropriate to clinical relevance but also limited false positives so that it becomes useful to the physician in a time-saving perspective. If considering PET-CT for screening of lung cancer as recurrently suggested [13, 22], a reliable tool with very high negative predictive values could save ample time at the corresponding imaging departments.

Specificity as a measure is difficult to assess in segmentation tasks since the definition of true negatives remains troublesome and would likely end up with very high numbers. For example, if each voxel in the lungs is defined as pathologic or not pathologic by the ground truth, then there would be disproportionally many true negative voxels compared to false positive voxels, leading to high specificity.

The lesion agreement in Table 1 show that the AI-model performs similarly with the readers, agreeing with the lesions that all readers agreed and marking additional lesions on its own similarly as reader B and C. Dice index showed, as expected, that even among readers the average score was not near-perfect (close to 1), and that the AI-model underperforms in this aspect.

A limitation in this study is the lack of external validation, e.g. examining the AI-tool’s performance on a data set from a different hospital; therefore, generalization of the tool has not been demonstrated.

**Fig. 7:**
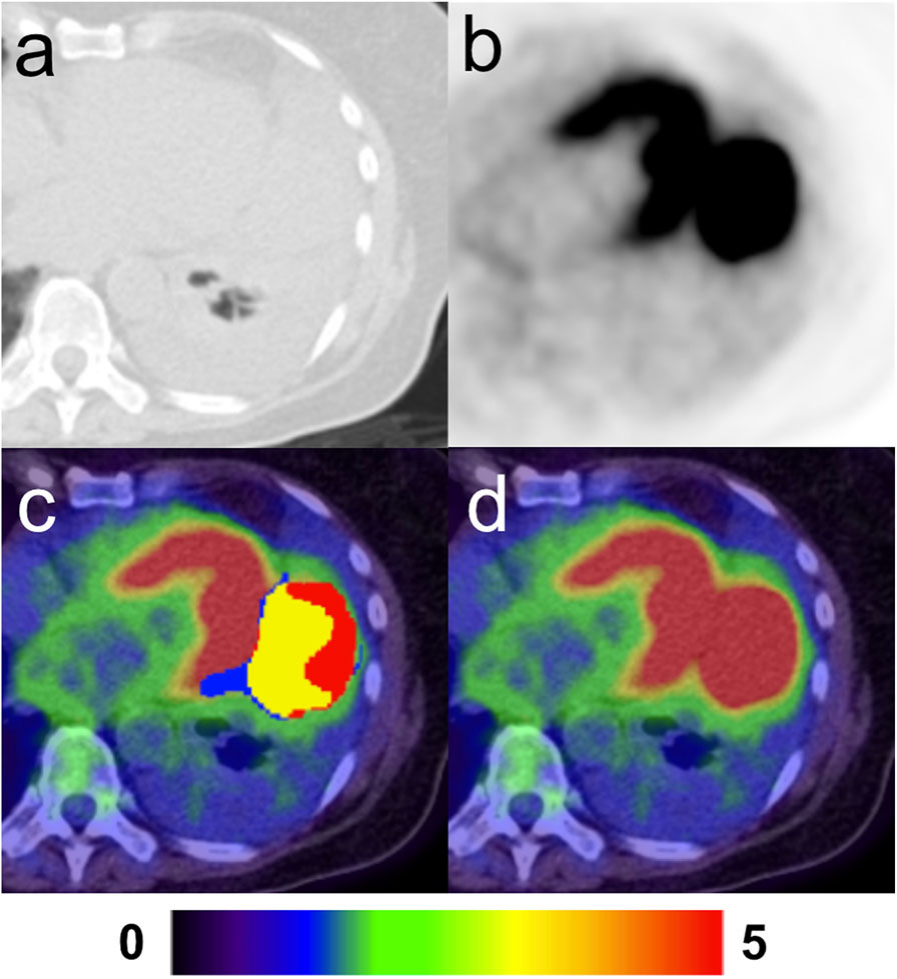
Large lesion in the left lung adjacent to the heart. **a** Axial CT with lung window. **b** Axial PET. **c** Fused axial PET-CT with overlaying segmentations; manual only (red), AI-only (blue) and manual + AI (yellow). **d** Fused axial PET-CT

Another limitation for the tool is medial lung lesions with or without concomitant high FDG-uptake in adjacent lymph node metastases or in the left ventricle of the heart (Fig. 7). Also, large lesions with complex necrotic components were difficult for the AI to segment correctly. Incorrect segmentation may result in over- or underestimation of TLG. There were five lesions with necrosis in the AI training group and in the test group there were four. It is unknown if a larger training group would have resulted in more accurate AI segmentation. These observations are in keeping with our results; the Bland-Altman analysis showed better agreement in TLG measurements for smaller lesions, which suggests that the AI-tool would perform at its best in a patient screening program, where findings of large tumours would likely be rare, or in the evaluation of indeterminate lung nodules.

Several meta-analyses have demonstrated the association of TLG with overall survival, in addition to patients with non-small cell lung cancer, in patients with head and neck cancer and lymphoma [23, 24]. An introduction of TLG as a prognostic biomarker in clinical practise will add quality to reports, facilitate clinical decision and effective patient care, but must be preceded by standardisation of automatic metabolic tumour volume and SUV_mean_ measurements as suggested by Barrington et al. [25].

The synergy between automatically localised and segmented lesions with, for example, automatically obtained biomarkers [14], radiomic features [26, 27] and even theragnostic [9] information could highly improve research in lung cancer.

Although multiple research groups are concomitantly working in AI-tools for FDG PET-CT to better understand a wide range of variables, a combined effort would undoubtedly have a vast influence in how AI could be used in a variety of cancers. One example could be the combined information obtained with AI-based tumour localisation and prognostic value of the volumetric prognostic index proposed by Zhang et al. [28].

We recognize that the small test group and the study being limited to one hospital are limitations in the current study. All FDG-avid lesions were not confirmed by biopsy, in particular lesions that were assessed as inflammatory, which is in keeping with clinical practise. Non-FDG-avid lesions were not present in our material but would certainly be missed if present in any AI-tool which is only based on PET-data. In a future study, we are planning on using AI-tools to assess lymph nodes and distant metastases outside of the thorax, there will be a larger sample size and another hospital added. The presented AI-method is naturally not ready to be introduced into clinical practice. The path to a clinical software, which is CE marked in Europe and FDA cleared in the USA, is long, but starts with feasibility studies such as this. Our AI-method is, however, available for other researchers (www.recomia.org), who are interested in giving valuable input to what eventually can become a clinically available AI-method.

## Conclusions

A completely automated AI-based method can be used to detect lung lesions with high sensitivity, but probably more important in the actual scenario is the very high negative predictive value reached on a patient basis, allowing specialists to focus on just positive cases to further investigate them. In future studies, we will also apply AI-methods for the assessment of lymph nodes and distant metastases. These types of clinical decision support tools appear to have significant clinical potential and are in study by multiple research groups.

## Data Availability

The datasets used and/or analysed during the current study are available from the corresponding author on reasonable request.

## Abbreviations

FDG: [^18^F]-fluorodeoxyglucose
PET-CT: Positron emission tomography with computed tomography
TLG: Total lesion glycolysis
AI: Artificial intelligence
CNN: Convolutional neural network
